# Transmission of Major and Minor Serum Proteins during Microfiltration of Skim Milk: Effects of Pore Diameters, Concentration Factors and Processing Stages

**DOI:** 10.3390/foods10040888

**Published:** 2021-04-18

**Authors:** Zhibin Li, Dasong Liu, Shu Xu, Wenjin Zhang, Peng Zhou

**Affiliations:** 1 State Key Laboratory of Food Science and Technology, Jiangnan University, Wuxi 214122, China; 6180112146@stu.jiangnan.edu.cn (Z.L.); 6180111077@stu.jiangnan.edu.cn (S.X.); 6180112118@stu.jiangnan.edu.cn (W.Z.); zhoupeng@jiangnan.edu.cn (P.Z.); 2 International Joint Research Laboratory for Functional Dairy Protein Ingredients, U.S.-China Dairy Innovation Center, Jiangnan University, Wuxi 214122, China

**Keywords:** microfiltration, serum proteins, bioactive proteins, membrane pore diameter, concentration factor, processing stage

## Abstract

Effects of pore diameters (100, 50, and 20 nm), concentration factors (1–8) and processing stages (1–5) on the transmission of major serum proteins (β-lactoglobulin and α-lactalbumin) and minor serum proteins (immunoglobulin (Ig) G, IgA, IgM, lactoferrin (LF), lactoperoxidase (LPO), xanthine oxidase (XO)) during ceramic microfiltration (MF) of skim milk were studied. Holstein skim milk was microfiltered at a temperature of 50 °C, a transmembrane pressure of 110 kPa and a crossflow velocity of 6.7 m/s, using a tubular single stainless steel module that consisted of three ceramic tubes, each with 19 channels (3.5 mm inner diameter) and a length of 0.5 m. For MF with 100 nm and 50 nm pore diameters, the recovery yield of major serum proteins in permeate was 44.3% and 44.1%, while the recovery yield of minor serum proteins was slightly less by 0%–8% than 50 nm MF. MF with 20 nm pore diameters showed a markedly lower (by 12%–45%) recovery yield for both major and minor serum proteins, corresponding with its lower membrane flux. Flux sharply decreased with an increasing concentration factor (CF) up to four, and thereafter remained almost unchanged. Compared to the decrease (88%) of flux, the transmission of major and minor serum proteins was decreased by 4%–15% from CF = one to CF = eight. With increasing processing stages, the flux gradually increased, and the recovery yield of both major and minor proteins in the permeate gradually decreased and reached a considerably low value at stage five. After four stages of MF with 100 nm pore diameter and a CF of four for each stage, the cumulative recovery yield of major serum proteins, IgG, IgA, IgM, LF, LPO, and XO reached 95.7%, 90.8%, 68.5%, 34.1%, 15.3%, 39.1% and 81.2% respectively.

## 1. Introduction

Although human milk is the best natural food for infant nutrition, sometimes infants do not get enough breast milk. Infant formula milk (IMF) is an ideal substitution [1]. The ratios of whey protein to casein are 20:80 and 60:40 in bovine and human milk, respectively. For the manufacture of infant formula, whey proteins are usually mixed with skim milk to increase their ratios relative to caseins [2]. The essential nutrition and biological functions of IMF are largely associated with whey proteins (WP). Besides the major constituents including β-lactoglobulin and α-lactalbumin, WP also contain minor bioactive components such as immunoglobulin (Ig) G, IgA, IgM, lactoferrin (LF), lactoperoxidase (LPO) and xanthine oxidase (XO) [3]. The major serum proteins in bovine milk are β-lactoglobulin (3.2–3.3 g/L) and α-lactalbumin (1.2–1.3 g/L), while the major serum proteins in human milk are α-lactalbumin (1.9–3.4 g/L). For the typical minor serum proteins including IgG, IgA, IgM and LF, bovine milk has a concentration around 0.15–0.8, 0.05–0.14, 0.04–0.1 and 0.02–0.5 mg/mL, respectively, while human milk has a concentration around 0.03, 0.96, 0.02 and 1.5–2.0 mg/mL [4]. These bioactive proteins show antibacterial, anti-pathogenic, anticarcinogenic and anti-inflammatory properties [5,6,7]. Some minor serum proteins are generally thermal sensitive and susceptible to heat-induced denaturation, and hence inactivation [8]. The commercial WP ingredients such as whey protein concentration and isolate are mainly derived from whey, the by-product of cheese making [9]. Bovine milk is heated and fermented during the cheese manufacturing process, and the obtained whey fractions are further heated to inactivate the remaining bacteria, thus changing the native structure of the WP [10]. Therefore, maintaining the native structure of bioactive serum proteins can improve the biological function of IMF.

Microfiltration (MF) is a pressure-driven separation process. MF can separate colloidal particles of different sizes [11]. The casein in bovine milk exists in the form of micelles with diameters ranging from 50 nm to 600 nm, while whey proteins generally have a size of about 2–5 nm [12,13,14]. Previous studies about the MF of skim milk mainly focused on the transmission of major serum proteins. Hurt and Barbano [15] investigated the effects of various processing factors on the MF of skim milk and found that the cumulative removal of major serum proteins was mainly affected by heat treatment of skim milk, concentration factor (CF) and processing stages (diafiltration steps). Jørgensen et al. [16] reported that ceramic membrane pore size and filtration temperature significantly affected serum protein separation from skim milk by MF, and the separation efficiency was optimal with a 100 nm membrane and at 50 °C. Schiffer et al. [17] reported that the separation of major serum proteins from skim milk was largely affected by the charged state of the membrane. However, there is still limited information regarding the effects of processing factors on the transmission of minor serum proteins during MF of skim milk.

Compared to whey fractions derived from cheese making, the serum fractions separated directly from skim milk by MF are clear and do not contain somatic cells, bacteria, bacteriophages, cheese fines or glycomacropeptide [11,18,19,20]. Infants fed with infant formula are prone to infections, gut inflammation and neurodevelopmental delays. One consideration is the repeated heat treatment encountered by the conventional WP ingredients such as demineralized whey powders and whey protein concentrate, resulting in decreased levels of multiple bioactive proteins (e.g., Ig and LF) and enzymes. The serum proteins derived directly from skim milk by MF (i.e., a nonthermal treatment) may serve as a better option than the conventional WP ingredients to provide protection against infection and inflammation in newborn infants, as their bioactive components would be better retained [21]. The serum proteins, especially the bioactive proteins and enzymes, generally maintain their native structure, and hence are suitable to be used as a protein ingredient to produce IMF. Optimal fractionation of serum proteins, especially the minor bioactive ones, from skim milk by MF, is of great interest to the IMF processors.

Therefore, this study compared the effects of pore diameters (100, 50, 20 nm), concentration factors (1–8) and processing stages (1–5) on the membrane flux as well as the transmission of major serum proteins and typical minor bioactive serum proteins and enzymes.

## 2. Materials and Methods

### 2.1. Materials

Fresh Holstein cow milk was purchased from Tianzi Dairy Co., Ltd. (Wuxi, Jiangsu, China) and then defatted (≤0.1 g/L, determined by ether extraction method of Chinese hygienic standard GB 5413.3–2010) using a CLARA 20LFCO cream separator (Alfa Laval Corp. AB, Lund, Sweden). Bacteria and somatic cells in skim milk were removed using a GCM-C-03 unit (Guochu Technology Co., Ltd., Xiamen, Fujian, China) equipped with three tubular ceramic membranes (1.4 μm pore diameter, 0.312 m^2^ surface area; TAMI Industries, Nyons, France) to obtain bacteria-removed milk (BRM). All other chemicals used were of analytical grade unless marked.

### 2.2. MF of Skim Milk with Different Pore Sizes, CF and Processing Stages

Microfiltration was performed at a temperature of 50 °C, a transmembrane pressure of 110 kPa and a crossflow velocity of 6.7 m/s, using the GCM-C-03 unit equipped with a tubular single stainless steel module consisting of 3 ceramic tubes, each with 19 channels (3.5 mm inner diameter) and a length of 0.5 m. BRM was pumped into the MF system described above and filtered using ceramic membranes at 50 °C with pore diameters of 100, 50 and 20 nm (0.312 m^2^ surface area; GC-CMF19/35/100S, GC-CUF19/35/50S, GC-CUF19/35/20S; Guochu Technology Co., Ltd., Xiamen, Fujian, China), respectively, and all the microfiltration was processed with CF = 3. Mean flux was calculated based on the accumulated permeate volume, membrane surface area and processing time using equation (1) as follows. Samuelsson et al. [22] proposed the empirical equation of flux = Re × 6.94 × 10^−10^ m/s as the best predictor for the limiting flux.

Re (Reynolds number) is expressed as: (*ρ*/μ) × υD

where ρ (1,018.2 kg/m^3^) is the density of skim milk, μ (0.00075 P·s) is the viscosity of skim milk, υ (6.7 m/s) is the cross-flow velocity of the feed and D (0.0035 m) is the inner diameter of the membrane channel [23,24]. 

Bacchin suggested that the critical flux is about two-thirds of the limiting flux [25]. The limiting and critical flux in the present study were calculated to be 80 L/m^2^ and 53 L/m^2^ per hour, respectively. The MF system in the present study was operated below the limiting flux to have a well capacity. Then, BRM was processed with the same MF system equipped with a 100 nm ceramic membrane to study the effects of CF on membrane flux and milk serum protein transmission. During the microfiltration, 100 mL permeate from the outlet of the MF system was collected when the CF reached 1× (initial), 2×, 3×, 4×, 5×, 6×, 7× and 8×. CF = 1 was assumed to be the initial processing point after the MF system was built up, i.e., when the feed inlet and outlet pressures reached a steady state. The transmission of serum proteins in permeate was calculated according to equation (2). Furthermore, BRM was microfiltered approximately at CF = 4 and 50 °C with a pore diameter of a 100 nm ceramic membrane. At the end of each processing stage, the retentate was diluted back to 1× using 50 °C reverse osmosis (RO) water. MF processes with 1, 2, 3, 4 and 5-stage (i.e., microfiltration and 4 subsequent steps of diafiltration) were carried out through the whole experiment. The MF permeate of each MF stage was collected for analysis, and the flux of every stage was represented as mean membrane flux.

Flux = V/(A × ∆t)(1)

where V (L) is the accumulated volume of permeate, A (m^2^) is the membrane surface area and Δt (h) is the corresponding processing time.

Transmission (%) = C_P_/C_R_ (2)

where C_P_ is the concentration of a specific component in the permeate and C_R_ is the concentration of the same component in the retentate.

The cleaning procedure of the MF system was completed according to Hurt et al. [26]. In brief, before each MF process, the storage solution, i.e., 2.5 g/L (*w/w*) NaHSO_3_, was flushed out of the MF system using RO water at 25 °C. The system was circulated with RO water at 60 °C for 5 min and then circulated with 10 g/L GCC-B01 alkaline membrane cleaning solution (Guochu, Technology Co., Ltd., Xiamen, Fujian, China) at 60 °C for 30 min. The system was cooled slowly (<10 °C/min) to 25 °C by step-wise exchange of RO water at 25 °C and then flushed with RO water at 25 °C until a neutral pH was reached. The permeate flux for RO water was measured at a transmembrane pressure of 170 kPa. The MF system was circulated with RO water at 80 °C for 30 min and then cooled to 50 °C by step-wise exchange of RO water at 25 °C. Then, the milk sample was added to the feed tank and MF was started. After finishing the MF treatment, the system was flushed with RO water at 25 °C until no retentate was visible and then circulated with 10 g/L GCC-B01 alkaline membrane cleaning solution and 0.55% (V/V) HNO_3_ solution at 60 °C for 30 min, respectively, with water flushing after each cleaning step until neutral pH was reached. The permeate flux for RO water was measured as mentioned above.

### 2.3. Major Serum Proteins Analysis

The protein profiles of permeate were determined using a Waters e2695 Separations Module (Waters Corp., Milford, MA, USA), equipped with a Waters XBridge BEH C4 column (250 mm × 4.6 mm 179 I.D.) and a Waters 2489 UV/Visible detector at 220 nm according to the method of Visser et al. [27]. The protein contents of permeate were calculated as a percentage of their peak area chromatogram relative to a BRM milk sample chromatogram.

A reducing SDS-page was conducted. Samples were diluted by a factor of 8 and then mixed with a loading buffer (25 mM Tris-HCl, 100 g/L glycerol (*v/v*), 20 g/L SDS (*w/v*), 50 g/L β-Mercaptoethanol (*v/v*) and 1 g/L bromophenol blue (*w/v*)) at 1:1. The mixed samples (10 μL) were loaded onto an SDS-PAGE gel (40 g/L acrylamide stacking and a 130 g/L acrylamide separating gel). The procedure of running, staining and de-staining for gels was completed according to Verdi et al. [28].

### 2.4. Minor Serum Proteins Analysis

Concentrations of IgG, IgA, IgM and LF were determined using ELISA test kits E10-118, E10-131, E10-101 and E10-126 (Bethyl Laboratories, Inc., Montgomery, TX, USA), respectively, based on the manufacturer’s protocols. Samples were diluted by a factor of 2000 for IgG, 500 for IgA, IgM and LF, using the dilutant solution E106 (Bethyl Laboratories, Inc.). The absorbance was measured using a Multiskan Sky spectrophotometer (Thermo Scientific, Waltham, MA, USA). A standard 4-parameter curve was fitted with the provided ELISA Quantitation Set Protocol using Soft-Max Pro software (Molecular Devices, LLC, San Jose, CA, USA).

LPO and XO activities were determined according to the method of Zou et al. [29,30] using a Cytation 5 imaging reader (BioTek Instruments, Inc., Winooski, VT, USA). 

### 2.5. Statistical Analysis

The experiments were triplicated, and data were analyzed using IBM SPSS Statistics 25 (IBM SPSS Statistics for Windows, IBM Corporation, Armonk, NY, USA). A Duncan test was used to assess the difference between means, and *p* < 0.05 was considered as statistically significant.

## 3. Results and Discussion

### 3.1. Effects of Pore Diameters on Recovery Yields of Major and Minor Serum Proteins

#### 3.1.1. MF Flux

**Figure 1:**
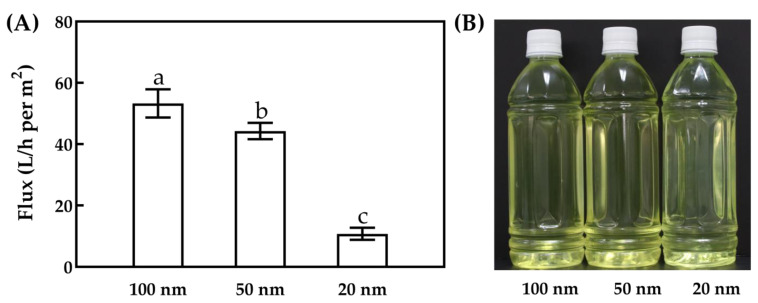
Membrane mean flux (**A**) and permeate photo (**B**) for MF with 100, 50 and 20 nm pore diameters. a–c, different lower-case letters indicate that the data differ significantly (*p* < 0.05). MF was performed at 50 °C, CF = 3, TMP = 110 kPa and crossflow velocity = 6.7 m/s. The permeate flux for RO water after cleaning was restored to more than 95% of that before each MF treatment.

Membrane flux was used to estimate the production efficiency of ceramic membranes. Membrane flux and photos of permeate with 100, 50 and 20 nm pore diameters during microfiltration processing are presented in Figure 1. As can be seen in Figure 1A, the flux decreased with the decrease of membrane pore diameter. In detail, the flux (*p* < 0.05) of a 100 nm pore diameter was five times higher than that of a 20 nm pore diameter, and was also slightly higher compared to a 50 nm pore diameter. The lower flux for MF with 20 nm pore diameter could be attributed to its smaller pore size. The flux value (53.29 L/m^2^ per h) of 100 nm was consistent with the experimental results reported by Adams and Barbano [31]. Although membrane flux of these three pore diameters was quite different, the photos (Figure 1B) of the three permeates from MF processing showed no significant difference. The possible explanation is that small colored molecules such as vitamin B could pass through all three membranes while casein could not [32].

#### 3.1.2. Major Serum Proteins

**Figure 2:**
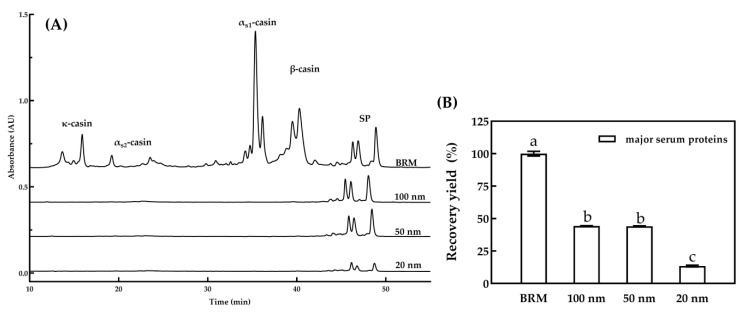
RP-HPLC chromatograms (**A**) and recovery yield (**B**) of major serum proteins (SP) in the permeate for MF with 100, 50 and 20 nm pore diameters. The recovery yield was calculated as a percentage of the mass of major serum proteins in the permeate relative to that in the BRM. a–c, different lower-case letters indicate that the data differ significantly (*p* < 0.05). MF was performed at CF = 3.

To analyze the influence of pore diameters on the recovery yield of major proteins, the protein profiles generated from RP-HPLC of permeate with different pore diameters are displayed in Figure 2A. The profiles showed that negligible amounts of caseins existed in the permeate from MF with 20, 50 and 100 nm membranes, which indicated that these membranes could separate serum proteins with the absence of caseins from skim milk by MF processing, in accordance with the results presented by Punidadas and Rizvi [33]. The recovery yield of major serum proteins in the permeate of different pore diameters is shown in Figure 2B. The recovery yield (*p* < 0.05) of serum proteins in the permeate of 20 nm was significantly lower than that of 50 nm and 100 nm. Compared with BRM, the recovery yield of 100 nm and 50 nm MF permeate was 44.3% and 44.1%, respectively. No significant difference (*p* ≥ 0.05) was found in these two groups. The result was lower than the 68% the recovery yield of serum proteins reported by Hurt et al. [26]. Jørgensen et al. [16] reported that the content of serum proteins in the permeate was higher at 100 nm compared to 50 nm. The differences in the serum protein content may be associated with practical operation conditions, especially the types of ceramic membrane. MF was completed using a standard cross-flow tubular ceramic membrane. Transmembrane pressure progressively decreased from the feed inlet of the membrane to the feed outlet of the membrane, resulting in a decreasing flux gradient over the length of the membrane, which caused rapid fouling at the inlet part of the membrane and hence underutilization of the outlet part [18,23]. Adams and Barbano [21] reported that MF performance could be improved by using a uniform transmembrane pressure system, graded permeability membrane or isoflux membrane, which could counteract the length-dependent defects and obtain a more homogenous flux and deposit layer formation along the membrane. This might explain the different recovery yields obtained by different membranes. The results showed that a 20 nm MF was not suitable for the separation of serum proteins and there was no difference between a 100 nm MF and a 50 nm MF in serum protein removal.

#### 3.1.3. Minor Serum Proteins

**Figure 3:**
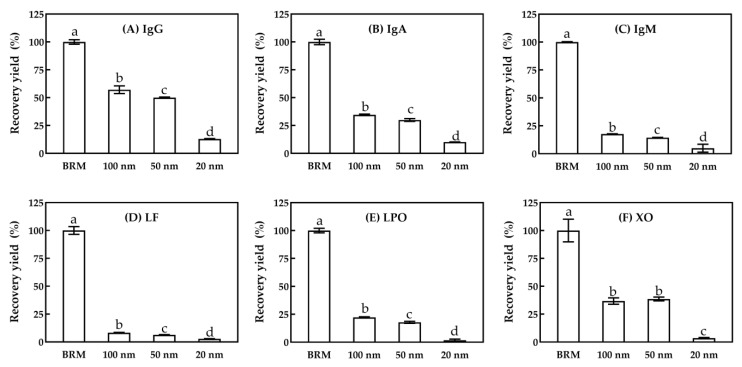
The recovery yield of native IgG (**A**), IgA (**B**), IgM (**C**) and LF (**D**), bioactive LPO (**E**) and XO (**F**) in permeate for MF with 100, 50 and 20 nm pore diameters. The recovery yield was calculated as a percentage of the mass of proteins or the total activity of enzymes in permeate relative to that in the BRM. a–d, different lower-case letters indicate that the data differ significantly (*p* < 0.05). MF was performed at CF = 3.

The recovery yields of native IgG, IgA, IgM, LF and bioactive LPO and XO in permeate with 100, 50 and 20 nm MF are shown in Figure 3. The recovery yield of IgG, IgA, IgM and LF in 100 nm MF permeate was 57.1%, 34.6%, 17.7% and 8.3% (*p* < 0.05), respectively. Each value was higher than that of the 50 nm permeate by 0%–8%. Similar trends could also be seen in the relative activity of LPO and XO, which was 22.2% and 36.8% in 100 nm MF permeate, respectively. The lowest recovery yield in the 20 nm MF indicated that the pore diameter of 20 nm MF was too small for the separation of bioactive proteins and enzymes. These results were perhaps caused by the difference in the native structures of IgG, IgA, IgM and XO. The forms of IgG, IgA and IgM are monomeric, dimeric and pentameric in milk, with hydrodynamic diameters of about 11, 18 and 24 nm, respective to particle size, leading to their decrease in recovery yields with the increase of pore diameters [14,34]. Native XO is also in dimeric form, and the molecular mass of XO (300 kDa) and IgM (385~430 kDa) is similar, leading to a similar tendency. [3,5]. Native LPO and LF are positively charged and hence bind partially to the negatively charged casein micelles in BRM, leading to a lower recovery yield in MF permeate as compared to native IgG, despite the former having a lower molecular mass of 70–80 and 78 kDa, as compared to that of 146–163 kDa for the IgG [35,36]. Therefore, considering the production efficiency as well as the recovery yield of major and minor serum proteins, a 100 nm ceramic membrane was selected for subsequent experiments.

### 3.2. Effects of CF on Transmission of Major and Minor Serum Proteins

#### 3.2.1. MF Flux

**Figure 4:**
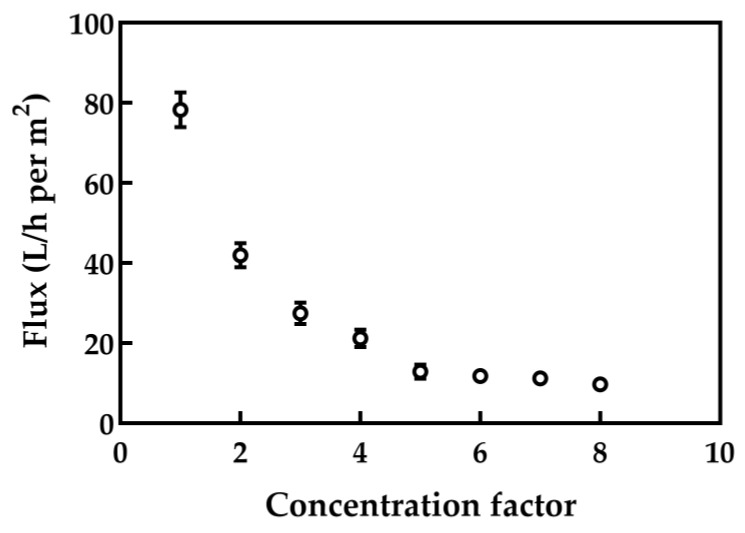
Membrane flux as a function of the CF for MF with a 100 nm pore diameter. The microfiltration was processed with TMP = 110 kPa, crossflow velocity = 6.7 m/s.

Figure 4 shows the change in membrane flux affected by CF during the microfiltration with a 100 nm ceramic membrane. The flux showed a decreased trend with the increase of CF; the flux dropped sharply when the CF was up to four, then the reduction rate slowed down. This result might be caused by the intensified polarization of protein concentrations, and the increased viscosity of retentate with the increase of the CF [37,38].

#### 3.2.2. Major Serum Proteins

**Figure 5:**
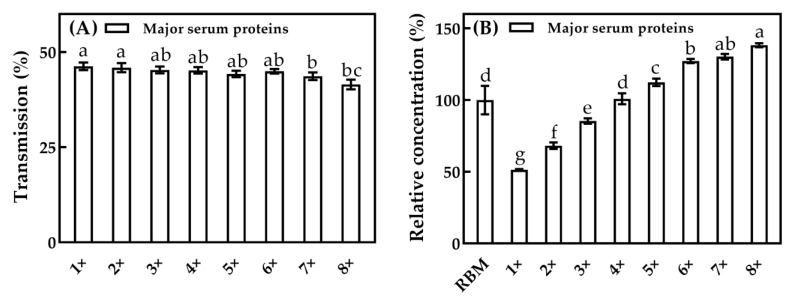
The transmission (**A**) and relative concentration (**B**) of major serum proteins in permeate at different CFs for MF with a 100 nm pore diameter. The relative value was calculated as a percentage of the concentration in the permeate relative to that in RBM. a–g, different lower-case letters indicate that the data differ significantly (*p* < 0.05).

The transmission (A) and relative concentration (B) of major serum proteins at different CFs for MF with a 100 nm pore diameter are shown in Figure 5. Between CF = one and CF = six, the transmission of major serum proteins was between 45.0%–46.3%, and no significant difference (*p* ≥ 0.05) was observed, while above CF = six, the transmission of major serum proteins decreased slightly (*p* < 0.05). The small particle size of serum proteins (2–5 nm) rendered their transmission through the 100 nm ceramic membrane limitedly affected by the concentration polariton phenomenon with the increase of the CF used in this present study. The relative concentration of major serum proteins in permeates increased gradually with the increase of the CF, which might be caused by the increased serum protein concentration in the retentate, as their transmission through the membrane was partially hindered [18]. In general, the transmission of major serum proteins is minimally affected by the increasing CF.

#### 3.2.3. Minor Serum Proteins

**Figure 6:**
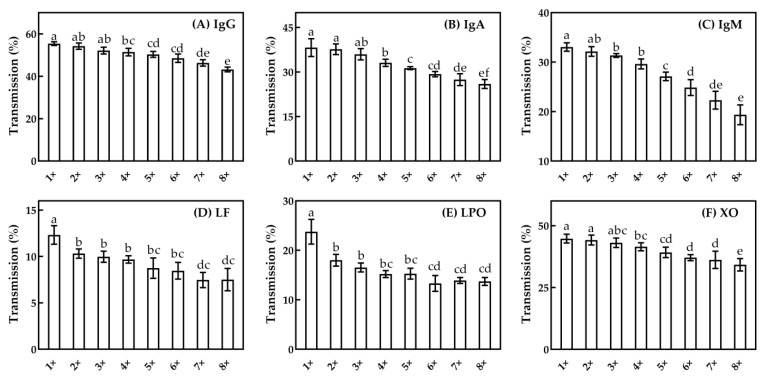
The transmission of native IgG (**A**), IgA (**B**), IgM (**C**), LF (**D**), bioactive LPO (**E**), and XO (**F**) in permeate at different CFs for MF with a 100 nm pore diameter. a–f: different lower-case letters indicate that the data differ significantly (*p* < 0.05).

The effects of CF on the transmission of native IgG, IgA, IgM and LF and the bioactive LPO and XO are shown in Figure 6. Generally, the transmission of minor serum proteins decreased with the increase of the CF. However, due to the different properties (e.g., size and charges) of minor serum proteins, their permeation through the membrane showed some differences in tendency. The highest transmission was observed for IgG, with the values being 55.4%–43.2% between CF one to eight. The transmission of IgA, IgM and XO was 38.2%, 33.1% and 44.8% at CF = one, respectively, and decreased to 25.9%, 19.4% and 34.2% when CF increased to eight. For IgA, IgM and XO, the transmission remained relatively stable between CF one to four, and then decreased gradually when CF was above four. As CF increased from one to eight, the transmission of LF and LPO was decreased from 23.8% to 13.7% and from 12.3% to 7.5%, respectively. The value of transmission for LF and LPO was lower than IgG, IgA, IgM and XO. In short, the order of transmission for all measured individua minor proteins was IgG > XO > IgA > IgM > LPO > LF. Generally, for IgG, XO, IgA and IgM, the ease of the transmission correlated inversely to their molecular size, while for LF and LPO, the transmission was further affected by their electrostatic binding to casein micelles given their isoelectric point around pH 8.7–9.6 [3,35]. Considering the change of MF flux and the transmission of major and minor serum proteins, especially IgM and IgA, CF = four was selected for subsequent experiments.

### 3.3. Effects of MF Stage on Recovery Yield of Major and Minor Serum Proteins

#### 3.3.1. MF Flux

**Figure 7:**
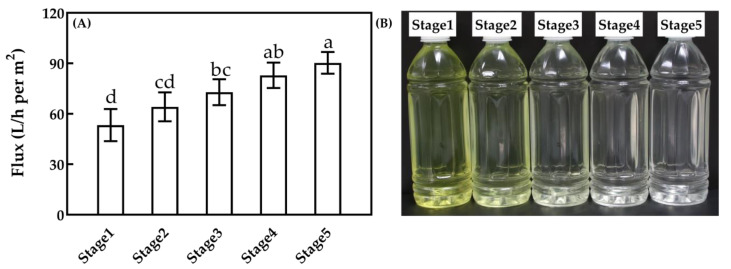
Membrane flux (**A**) and permeate photo (**B**) for 100 nm MF at different processing stages. a–d, different lower-case letters indicate that the data differ significantly (*p* < 0.05).

The membrane flux (A) and permeate photo (B) for 100 nm MF at different processing stages are shown in Figure 7. The flux (*p* < 0.05) was 53.3, 64.2, 72.8, 82.9 and 90.3 L/h per m^2^ at each stage, which presented an upward trend overall. The color of MF permeate became shallower as the MF stage increased. The concentration of soluble components and feed viscosity of BRM decreased with the increase of the processing stage [31], which led to the increase of MF flux. At the same time, the colored components, such as riboflavin, were gradually washed out in the BRM. Therefore, the color of the MF permeated liquid got increasingly lighter.

#### 3.3.2. Major Serum Proteins

**Figure 8:**
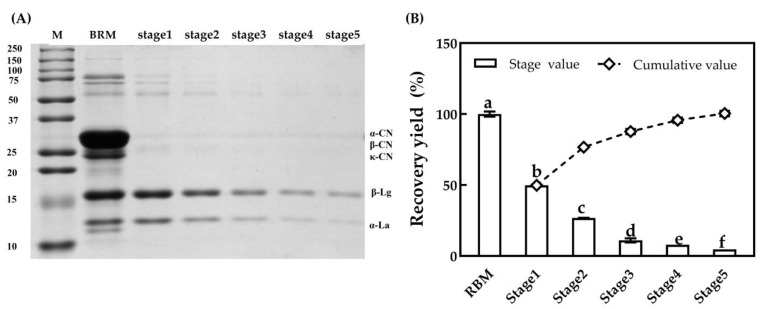
SDS-PAGE patterns of permeate (**A**) and the recovery yield (**B**) of major serum proteins in permeate for 100 nm MF at different processing stages. a–f, different lower-case letters indicate that the data differ significantly (*p* < 0.05).

The SDS-PAGE gel image of permeate at different MF stages is shown in Figure 8A. The samples of permeates were prepared in the same dilution, multiplied, and loaded at the same level. The bands of β-lactoglobulin and α-lactalbumin gradually became lighter as MF stages increased, and the bands of casein were not observed, indicating that there was little casein passing through the 100 nm ceramic membrane, which is in agreement with the results reported by Adams and Barbano [31]. The recovery yields of major serum proteins at different MF stages are presented in Figure 8B. The recovery yield of major serum proteins had significant differences for each MF stage (*p* < 0.05), which was 49.9%, 26.9%, 11.1%, 8.0% and 4.7%, respectively. Adams et al. [31] reported that it would take seven stages to remove 95% of the serum proteins from skim milk with CF = three by a 100 nm ceramic membrane, while it only needed four stages with CF = four in this study. However, the recovery yield of major serum proteins was lower compared to the study by Hurt et al. [26], which might be related to the differences in ceramic membranes, despite the same pore diameter. MF efficiency was closely associated with membrane type and processing conditions [18]. Unlike MF with a uniform transmembrane pressure, such as when using a graded permeability membrane, the MF with a standard membrane as applied in this present study would cause a decreasing flux gradient along the membrane and hence rapid fouling at the inlet part as well as underutilization of the outlet part of the membrane [10,31]. Generally, a higher temperature (i.e., 45–55 °C), a moderately higher cross-flow velocity and a higher transmembrane pressure below the value, corresponded to limiting flux and a higher MF efficiency [39]. Adams et al. reported different recovery yields of serum proteins with ceramic MF when using ceramic membranes from different types [31]. The recovery yield of major serum proteins gradually decreased because the content of major serum proteins in the retentate decreased as the processing stage increased.

#### 3.3.3. Minor Serum Proteins

**Figure 9:**
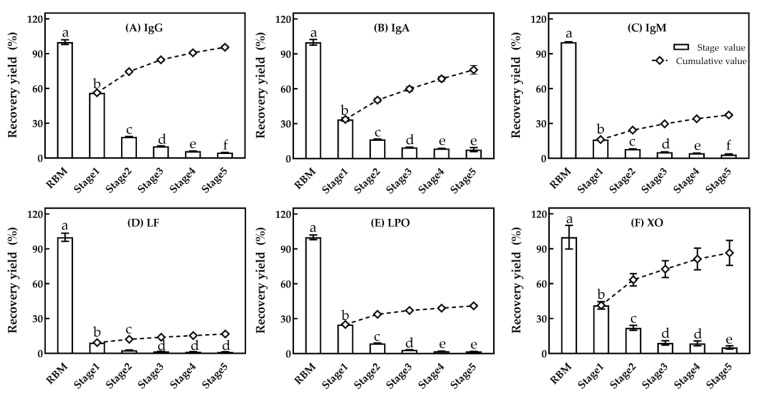
The recovery yield of native IgG (**A**), IgA (**B**), IgM (**C**) and LF (**D**), and bioactive LPO (**E**) and XO (**F**) in permeate for 100 nm MF at different processing stages. a–f, different lower-case letters indicate that the data differ significantly (*p* < 0.05).

Figure 9 shows the recovery yield of native IgG (A), IgA (B), IgM (C), LF (D), bioactive LPO (E) and XO (F) in permeate with a 100 nm ceramic membrane for each stage of a five-stage MF processing. There was a tendency that minor serum proteins in permeate decreased as the processing stage increased (*p* < 0.05), especially in stage five. The recovery yield of bioactive IgG, IgM, LF, LPO, XO was only 1%–4%, and for IgA, the recovery yield was 7%. The cumulative recovery yield of native IgG, IgA, IgM, LF and bioactive LPO and XO in permeate of four-stage processing was 90.8%, 68.5%, 34.1%, 15.3%, 39.1% and 81.2%, respectively. Lower (*p* < 0.05) recovery yields of bioactive LF and LPO from five-stage MF processing indicated that some of these were kept in retentate, which might be caused by LF and LPO binding partially to the negatively charged casein micelles, and thus being hardly affected by the MF stages. This result was consistent with that previously reported by Heidebrecht [14]. Native IgM is a polymeric immunoglobulin and a pentameric form in bovine milk with an oblate-like shape [34], which might contribute to the comparatively lower contents than IgG in permeates. Schiffer et al. [17] reported that MF performance was associated with membrane charge, besides the nominal pore size and the width of the pore size distribution. Therefore, considering the MF flux and the recovery yields of major and minor serum proteins, four-stage MF processing was selected to produce serum proteins.

## 4. Conclusions

For MF with 100 nm and 50 nm pore diameters, the recovery yield of major serum proteins in permeate was 44.3% and 44.1%, while the recovery yield of minor serum proteins was slightly smaller by 1%–8% than a 50 nm MF. MF with 20 nm pore diameters showed a markedly lower (by 12%–45%) recovery yield of both major and minor serum proteins, corresponding with its lower membrane flux. Flux sharply decreased with an increasing concentration factor (CF) up to four, and thereafter remained almost unchanged. Compared to the decrease (88%) of flux, the transmission of major and minor serum proteins decreased by 4%–15% from CF = one to CF = eight. The order of transmission for all measured individual minor proteins was IgG > XO > IgA > IgM > LPO > LF. With increasing processing stages, the flux also gradually increased, and the concentration of both major and minor proteins in the permeate gradually decreased and reached a considerably low value at stage five. After four stages of MF with 100 nm pore diameter and a CF of four for each stage, the cumulative recovery yield of major serum proteins, IgG, IgA, IgM, LF, LPO, and XO reached 95.7%, 90.8%, 68.5%, 34.1%, 15.3%, 39.1% and 81.2% respectively. Within the ranges of the three factors studied, serum proteins were produced using MF with 100-nm, CF = four and four stages.

## Data Availability

The data presented in this study are available on request from the corresponding author.
